# Fingerprints of slingshot non-sequential double ionization on two-electron probability distributions

**DOI:** 10.1038/s41598-019-55066-1

**Published:** 2019-12-11

**Authors:** G. P. Katsoulis, A. Emmanouilidou

**Affiliations:** Department of Physics and Astronomy, University College London, Gower Street, London, WC1E 6BT United Kingdom

**Keywords:** Atomic and molecular interactions with photons, Attosecond science

## Abstract

We study double ionization of He driven by near-single-cycle laser pulses at low intensities at 400 nm. Using a three-dimensional semiclassical model, we identify the pathways that prevail non-sequential double ionization (NSDI). We focus mostly on the delayed pathway, where one electron ionizes with a time-delay after recollision. We have recently shown that the mechanism that prevails the delayed pathway depends on intensity. For low intensities slingshot-NSDI is the dominant mechanism. Here, we identify the differences in two-electron probability distributions of the prevailing NSDI pathways. This allows us to identify properties of the two-electron escape and thus gain significant insight into slingshot-NSDI. Interestingly, we find that an observable fingerpint of slingshot-NSDI is the two electrons escaping with large and roughly equal energies.

## Introduction

Non-sequential double ionization (NSDI) of atoms driven by intense laser fields is a fundamental process which has attracted considerable theoretical and experimental interest^1–23^. The three-step model underlies NSDI^1^. First, one electron tunnels through the field-lowered Coulomb potential. This tunnel-ionizing electron can return to the core and transfer energy to the other electron. For high intensities, the main pathway of NSDI is the direct one. The recolliding electron transfers energy to the other electron that suffices for both electrons to ionize following recollision. For low intensities, the delayed pathway of NSDI prevails. The recolliding electron transfers energy to the bound electron, however, this energy is sufficient for only one of the two electrons to ionize following recollision. As a result of the energy transferred, the other electron transitions to an exited state of the remaining ion.

Until recently, the delayed pathway of NSDI was generally accepted as being equivalent to recollision-induced excitation with subsequent field ionization (RESI)^7,22^. According to RESI, the electron that transitions to an excited state after recollision, ionizes later in time at extrema of the laser field. It does so mainly with the assistance of the field. However, recently, we have shown that RESI is not always the most important mechanism of the delayed pathway of NSDI. We did so by employing a three-dimensional semiclassical model^24^. Several classical and semiclassical models have been employed in recent years to describe processes in strong fields^23,25–27^, for a review see ref. ^28^. When He is driven by near-single-cycle pulses of 400 nm wavelength, we have shown that a new mechanism overtakes RESI in the delayed pathway of NSDI^29^, at small intensities below the recollision threshold. We labeled this mechanism slingshot-NSDI. We have shown that in slingshot-NSDI, following the transition of an electron to an excited state, due to the attractive force that the nucleus exerts to this electron, the electron subsequently undergoes a Coulomb slingshot motion. This slingshot motion we identify in the context of Coulomb forces is reminiscent of the slingshot motion, in the context of gravitational forces, that a spacecraft undergoes altering its motion around a planet. During Coulomb slingshot the electron ionizes mostly around the second extremum of the field with the assistance of both the nucleus and the laser field. Due to the electron that ionizes last undergoing slingshot motion, the two electrons escape opposite to each other along the direction of the laser field. This anti-correlated two-electron escape has previously been attributed to multiple recollisions, a mechanism that was put forth in the context of RESI^30–34^. This pattern of two-electron escape has been found to prevail NSDI of several atoms driven by intense long duration pulses. It has been the object of many theoretical and experimental studies^30–37^. Hence, slingshot-NSDI offers an alternative explanation to multiple recollisions.

Here, we show that anti-correlated two-electron escape is not the only feature that distinguishes slingshot-NSDI from the other NSDI pathways. The main NSDI pathways, which are considered in the current study, are the direct pathway, RESI and the double delayed pathway. In the latter pathway both electrons ionize with a delay following recollision. We show that slingshot-NSDI has very distinct fingerprints in both energy and angular two-electron probability distributions. These features can be observed experimentally. Hence, this study paves the way for identifying slingshot-NSDI by kinematically complete experiments that employ near-single-cycle pulses where control of carrier envelope phase (CEP) is achieved^34,38–40^.

## Method

We consider He driven by a near-single-cycle laser pulse at intensities 5 × 10^14^ W/cm^2^ and 7 × 10^14^ W/cm^2^ at 400 nm. Both intensities are below the recollision threshold, which corresponds to an intensity of 8.6 × 10^14^ W/cm^2^. The latter corresponds to the maximum energy of the electron returning to the core being equal to the energy needed to transition to the first excited state of the remaining ion. The maximum energy of the electron returning to the core is 3.17 ${ {\mathcal E} }_{0}^{2}\mathrm{/(4}{\omega }^{2})$^1^, which is equal to 23.7 eV at 5 × 10^14^ W/cm^2^ and 33.1 eV at 7 × 10^14^ W/cm^2^; ${ {\mathcal E} }_{0}$ and *ω* are the strength and frequency of the field.

We use a laser field of the form

1 $$\overrightarrow{ {\mathcal E} }({\rm{t}})={ {\mathcal E} }_{0}\,\exp (-2\,\mathrm{ln}\,2{(\frac{{\rm{t}}}{\tau })}^{2})\cos (\omega {\rm{t}}+\phi )\hat{z},$$

where *ϕ* is the CEP, and *τ* = 2 fs is the full-width-half-maximum of the pulse duration in intensity. We employ atomic units, unless otherwise stated.

We use a three-dimensional (3D) semiclassical model that is formulated in the framework of the dipole approximation^24^. Previous successes of this model include verifying that electron backscattering from the nucleus accounts for the finger-like structure in NSDI of He driven by long laser pulses at higher intensities^24^. This finger-like structure was predicted theoretically^14^ and obtained experimentally^15,16^. Moreover, it was explained in a classical framework^24,41^. In addition, using this 3D model, we investigated the direct versus the delayed pathway of NSDI for He driven by a long duration laser pulse at 400 nm^42^. For intensities ranging from below- to above-the-recollision threshold, we achieved excellent agreement with fully ab-initio quantum mechanical calculations. In addition, using this model we obtained very good agreement with experimental results for several observables of NSDI for Ar when driven by near-single-cycle laser pulses at 800 nm^43^. These observables were obtained as a function of CEP for intensities ranging from below- to above-the-recollision threshold.

In this model, one electron (recolliding) tunnel-ionizes through the field-lowered Coulomb-barrier with a tunnel-ionization rate that is described by the quantum mechanical Ammosov-Delone-Krainov (ADK) formula^44,45^. We select the tunnel-ionization time, t_0_, using importance sampling^46^ in the time interval the field is present, that is, [−2*τ*, 2*τ*]. The importance sampling distribution is given by the ADK ionization rate. The exit point of the recolliding electron is along the laser-field direction and is computed using parabolic coordinates^47^. The electron momentum is taken to be equal to zero along the laser field. The transverse momentum is given by a Gaussian distribution which represents the Gaussian-shaped filter with an intensity-dependent width arising from standard tunneling theory^45,48,49^. The initially bound electron is described by a microcanonical distribution^50^. The weight of each classical trajectory i that we propagate in time is given by

2 $${{\rm{W}}}_{{\rm{i}}}={{\rm{W}}}_{{\rm{i}}}^{1}\cdot {{\rm{W}}}_{{\rm{i}}}^{2},$$

where

3 $${{\rm{W}}}_{{\rm{i}}}^{1}\propto {(\frac{1}{|\overrightarrow{ {\mathcal E} }({{\rm{t}}}_{0})|})}^{{{\rm{2n}}}^{\ast }-1}\exp (-\frac{2\,{\kappa }^{3}}{\mathrm{3|}\overrightarrow{ {\mathcal E} }({{\rm{t}}}_{0})|})$$

is the ADK ionization rate^44,45^ at the time t_0_ of tunnel-ionization. The effective principal quantum number, n^*^, is given by ${{\rm{I}}}_{{{\rm{p}}}_{1}}={{\rm{Z}}}^{2}{/\text{2n}}^{\ast 2}$, while $\kappa =\sqrt{2{{\rm{I}}}_{{{\rm{p}}}_{1}}}$ and I_p1_ is the first ionization potential. The weight for electron 1 to have a transverse velocity equal to v_⊥_ at the time t_0_ is denoted by ${{\rm{W}}}_{{\rm{i}}}^{2}$ and is given by

4 $${{\rm{W}}}_{{\rm{i}}}^{2}\propto \frac{{{\rm{v}}}_{\perp }}{|\overrightarrow{ {\mathcal E} }({{\rm{t}}}_{0})|}\exp (-\frac{{{\rm{v}}}_{\perp }^{2}{\rm{\kappa }}}{|\overrightarrow{ {\mathcal E} }({{\rm{t}}}_{0})|}).$$

Once the initial conditions are specified at time t_0_, the position and momentum of each electron are propagated classically in time. We do so using the three-body Hamiltonian of the two electrons with the nucleus kept fixed. All Coulomb forces are accounted for: the interaction of each electron with the nucleus and the laser field and the electron-electron interaction are all included in the time propagation. We also account for the Coulomb singularity by using regularized coordinates^51^. During the time propagation each electron is interacting with the nucleus with charge Z = 2. A trajectory is labeled as a doubly-ionized event if asymptotically, i.e. t → ∞, the energies of both electrons are positive. The double ionization probability is given by

5 $${{\rm{P}}}_{{\rm{DI}}}=\frac{{\sum }_{{\rm{i}}}^{{{\rm{N}}}_{{\rm{DI}}}}{{\rm{W}}}_{{\rm{i}}}}{{\sum }_{{\rm{i}}}^{{\rm{N}}}{{\rm{W}}}_{{\rm{i}}}}$$

where N_DI_ and N are the numbers of doubly-ionized and all events, respectively.

We identify the main pathways of energy transfer in each double ionization event. To do so we compute the time difference between the recollision time t_rec_ and the ionization time t_i_ of each electron, with i = 1, 2. We label the electron that ionizes first as electron 1 and the one that ionizes last as electron 2. To identify these times, for each classical trajectory, we compute the pair potential energy and obtain its maximum which corresponds to the time of minimum approach of the two electrons. We define this time of minimum approach as the recollision time. Moreover, we define the ionization time for each electron, t_i_, as the time when the compensated energy ${(p}_{x,i}^{2}+{{\rm{p}}}_{y,i}^{2}+{{(p}_{z,i}-{\mathscr A}(t))}^{2})/2-{Z/r}_{{\rm{i}}}$ becomes positive and remains positive thereafter^52^, with i = 1, 2 and ${{\rm{p}}}_{{\rm{i}}}={{\rm{p}}}_{x,i}\hat{{\rm{x}}}+{{\rm{p}}}_{y,i}\hat{{\rm{y}}}+{{\rm{p}}}_{z,i}\hat{{\rm{z}}}$; ${\mathscr A}({\rm{t}})$ is the vector potential and *Z* = 2. We compare the time difference between the recollision time and the ionization time of each electron with the time interval t_diff_ where the electron pair potential energy undergoes a sharp change due to recollision. For the laser field intensities considered in this work, we find t_diff_ to be roughly equal to 1/8 laser cycle (T). We list in Table 1 the conditions satisfied by the direct, delayed or double delayed double ionization events. We find that in the delayed pathway, the probability for electron 2 to be the recolliding electron increases with decreasing intensity.

**Table 1:** Conditions for energy transfer double ionization pathways.

Δt_1_ = t_1_ − t_rec_ & Δt_2_ = t_2_ − t_rec_		
direct	delayed	double delayed
$$\begin{array}{lll}{\Delta t}_{2} & < & {{\rm{t}}}_{{\rm{diff}}}\\ {{\rm{t}}}_{1} & < & {{\rm{t}}}_{2}\end{array}$$	$$\begin{array}{lll}{\Delta t}_{1} & < & {{\rm{t}}}_{{\rm{diff}}}\\ {\Delta t}_{2} & > & {{\rm{t}}}_{{\rm{diff}}}\end{array}$$	$$\begin{array}{lll}{\Delta t}_{1} & > & {{\rm{t}}}_{{\rm{diff}}}\\ {\Delta t}_{2} & > & {{\rm{t}}}_{{\rm{diff}}}\end{array}$$

For the results presented in this work, we consider the intensities 5 × 10^14^ W/cm^2^ and 7 × 10^14^ W/cm^2^. For both intensities, 12 CEPs are considered ranging from $\phi ={0}^{\circ }$ to $\phi ={330}^{\circ }$ in steps of 30°. For each *ϕ*, at 7 × 10^14^ W/cm^2^ we obtain roughly 10^4^ doubly-ionized events as a result of running 500, 12-hour jobs, while at 5 × 10^14^ W/cm^2^ we obtain 5 × 10^3^ doubly-ionized events as a result of running 4000 jobs, 12-hour jobs; one job corresponds to 1 CPU. The double ionization probability is 2.7 × 10^−6^ at 5 × 10^14^ W/cm^2^ and 6.3 × 10^−5^ at 7 × 10^14^ W/cm^2^. For the results presented regarding total double ionization the average has been taken over all CEPs for each intensity.

## Results

### Slingshot-NSDI

First, we identify the prevailing pathways of double ionization when He is driven by a 2 fs laser pulse at 400 nm and at intensities of 5 × 10^14^ W/cm^2^ and 7 × 10^14^ W/cm^2^. We find that the pathways prevailing NSDI ionization are the delayed and the direct ones at 7 × 10^14^ W/cm^2^ and the delayed and the double delayed ones at 5 × 10^14^ W/cm^2^. For the delayed pathway, we note a significant change that takes place with decreasing intensity. The mechanism that prevails switches from RESI to slingshot-NSDI. Since both RESI and slingshot-NSDI are mechanisms of the delayed pathway, in both mechanisms electron 1 ionizes soon after recollision and electron 2 transitions to an excited state of He^+^. Slingshot-NSDI and RESI differ in the mechanism that underlies the subsequent ionization of electron 2. In RESI this mechanism involves electron 2 ionizing with the assistance of the laser field at later times at extrema of the field. In contrast, in slingshot-NSDI we find that electron 2 ionizes with the assistance of both the nucleus and the laser field. Next, we briefly outline some of the properties of slingshot-NSDI^29^.

**Figure 1:**
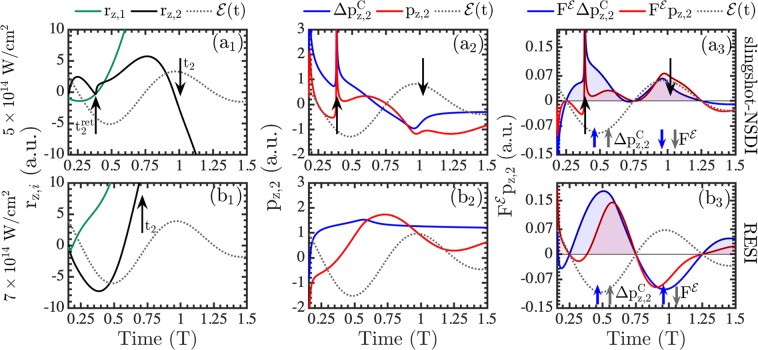
Slingshot-NSDI versus RESI. Slingshot-NSDI at 5 × 10^14^ W/cm^2^ (**a**) and RESI at 7 × 10^14^ W/cm^2^ (**b**). We plot as a function of time (**a1**) r_z,1_ and r_z,2_ (**a2**) p_z,2_ and ${\Delta p}_{z,2}^{{\rm{C}}}$ and (**a3**) F${}^{ {\mathcal E} }\,{{\rm{p}}}_{z,2}$ and F${}^{ {\mathcal E} }\,{\Delta p}_{z,2}$. Coulomb slingshot is enclosed by the black arrows, with the up and down arrow depicting p_z,2_ being along the +$\hat{z}$-axis and −$\hat{z}$-axis, respectively, at the start and the end of the Coulomb slingshot motion. The beginning of the time axis is t_rec_.

In particular, Fig. 1(a1) shows that in slingshot-NSDI electron 2 undergoes a Coulomb slingshot motion around the nucleus, see motion enclosed by the black arrows in Fig. 1(a1). In Fig. 1, we consider a double ionization event corresponding to CEP equal to zero. Similar results hold for all CEPs, while if the re-colliding electron tunnel-ionizes at a minimum (maximum) of the field, the direction of escape of electrons 1 and 2 is the same (reversed) as in Fig. 1(a1). As a result of the slingshot motion, electron 2 escapes opposite to electron 1 and its momentum, p_*z*,2_, undergoes a large change. The momentum of electron 2 is large and points along the direction of the force from the laser field at the start and at the end of the slingshot motion, see Fig. 1(a2). We show that the momentum of electron 2 changes by a large amount due to the nucleus. To show that this is the case, we sum the momentum changes due to the interaction with the nucleus and electron 1, ${\Delta p}_{z,2}^{{\rm{C}}}$, and with the laser field, ${\Delta p}_{z,2}^{ {\mathcal E} }$ to obtain the total momentum, p_*z*,2_, as follows:

6a $${{\rm{p}}}_{z,2}(t)={{\rm{p}}}_{z,2}{(t}_{0})+{\Delta p}_{z,2}^{{\rm{C}}}{(t}_{0}\to t)+{\Delta p}_{z,2}^{ {\mathcal E} }{(t}_{0}\to t)$$

6b $${\Delta p}_{z,2}^{{\rm{C}}}(t)={\int }_{{{\rm{t}}}_{0}}^{{\rm{t}}}\,(\frac{-{\rm{Z}}\,{{\rm{r}}}_{z,2}}{{|r}_{2}{|}^{3}}+\frac{{{\rm{r}}}_{z,2}-{{\rm{r}}}_{z,1}}{|{{\bf{r}}}_{2}-{{\bf{r}}}_{1}{|}^{3}})d{\rm{t}}^{\prime} $$

6c $${\Delta p}_{z,2}^{ {\mathcal E} }(t)={\mathscr A}({\rm{t}})-{\mathscr A}({{\rm{t}}}_{0}\mathrm{)}.$$

In the delayed pathway, following recollision, the Coulomb interaction between the two electrons is very small. Thus, the repulsive force between the two electrons contributes only a constant term to ${\Delta p}_{z,2}^{{\rm{C}}}$. In Fig. 1(a2), we plot how the momentum of electron 2 changes as a function of time due to the nucleus. One easily concludes that, during the Coulomb slingshot motion, the term ${\Delta p}_{z,2}^{{\rm{C}}}$ contributes the most to the sharp change of the total momentum of electron 2.

Next, we show that, during the slingshot motion, the laser field provides sufficient energy to electron 2 to ionize due to the large change in the electron momentum. Shortly after recollision, at time t_init_ = t_rec_ + t_diff_, the repulsive force between the two electrons is roughly zero. Hence, after this time, it is a very good approximation to assume that only the force of the laser field acts on electron 2. As a result, the work done by the laser field is mainly responsible for the change in the energy of electron 2, which can be expressed as follows:

7 $$H(t)=\frac{{{\rm{p}}}_{2}{{(t}_{{\rm{init}}})}^{2}}{2}-\frac{{\rm{Z}}}{{{\rm{r}}}_{2}{(t}_{{\rm{init}}})}+{\int }_{{{\rm{t}}}_{{\rm{init}}}}^{{\rm{t}}}\,{{\rm{F}}}^{ {\mathcal E} }{{\rm{p}}}_{z,2}d{\rm{t}}^{\prime} ,$$

where the laser field force is denoted by ${{\rm{F}}}^{ {\mathcal E} }(t)=-\, {\mathcal E} (t)$, while ${{\rm{F}}}^{ {\mathcal E} }{{\rm{p}}}_{z,2}$ denotes the rate of change of the energy of electron 2. At the start of the Coulomb slingshot motion, shortly after the laser field reaches a zero value, electron 2 and the nucleus come close to each other at ${{\rm{t}}}_{2}^{{\rm{ret}}}$. At the start of this slingshot motion the momentum of electron 2 and the force from the laser field have the same direction along the +$\hat{z}$-axis. The end of the slingshot motion takes place roughly half a laser cycle later, in the time interval [0.75, 1.25]T. At the end of slingshot, the momentum of electron 2 and the force of the laser field have the same direction, which is along the −$\hat{z}$-axis. Hence, during the Coulomb slingshot, the rate of change of the energy of electron 2 due to the laser field, i.e. ${{\rm{F}}}^{ {\mathcal E} }{{\rm{p}}}_{z,2}$, is mostly positive in the first half cycle [0.25, 0.75]T and the second one [0.75, 1.25]T after recollision. These positive values are denoted by the red-shaded area in Fig. 1(a3). Figure 1(a3) clearly shows that ${{\rm{F}}}^{ {\mathcal E} }{{\rm{p}}}_{z,2}$ is positive due to ${{\rm{F}}}^{ {\mathcal E} }{\Delta p}_{z,2}^{{\rm{C}}}$ being positive, see blue-shaded area in Fig. 1(a3). Thus, the large change in the momentum of electron 2 due to Coulomb slingshot is the reason that the work provided to electron 2 by the laser field is positive. This leads to ionization of electron 2 around the second extremum of the field after recollision, i.e. in the time interval [0.75, 1.25]T. In contrast, in RESI electron 2 can ionize at any extremum of the laser field.

In Fig. 1(b), we plot a trajectory corresponding to the RESI mechanism, which dominates the delayed pathway at 7 × 10^14^ W/cm^2^, and compare it to the trajectory corresponding to the slingshot-NSDI mechanism at 5 × 10^14^ W/cm^2^, plotted in Fig. 1(a). In RESI, following re-collision, electron 1 escapes from the core in a similar manner as in slingshot-NSDI, see Fig. 1(b1). However, compared to slingshot-NSDI in Fig. 1(a1), electron 2 escapes to the continuum faster and as a result the interaction with the nucleus is much smaller. This is demonstrated, after re-collision, by an almost constant momentum change of electron 2 due to the nucleus, see blue line in Fig. 1(b2). Hence, after re-collision, the laser field is mainly responsible for the change in the total momentum of electron 2, see red line in Fig. 1(b2). That is, the most important contribution to p_z,2_ for the RESI mechanism is $\Delta {p}_{z\mathrm{,2}}^{ {\mathcal E} }$ (not shown), while for the slingshot-NSDI is ${\Delta p}_{z,2}^{{\rm{C}}}$, see Fig. 1(a2).

In contrast to slingshot-NSDI at 5 × 10^14^ W/cm^2^ where ${{\rm{F}}}^{ {\mathcal E} }{\Delta p}_{z,2}^{{\rm{C}}}$ is positive in the first half cycle [0.25, 0.75]T and the second one [0.75, 1.25]T after recollision, for RESI at 7 × 10^14^ W/cm^2^, ${{\rm{F}}}^{ {\mathcal E} }{\Delta p}_{z,2}^{{\rm{C}}}$ is positive only in the first half-cycle, see blue-shaded area in Fig. 1(b3). However, at 7 × 10^14^ W/cm^2^ the strength of the laser field ${F}^{ {\mathcal E} }$ is larger. Therefore, in the first half cycle, the rate of change of the energy of electron 2, ${{\rm{F}}}^{ {\mathcal E} }{{\rm{p}}}_{z,2}$, has larger positive values at 7 × 10^14^ W/cm^2^ rather than at 5 × 10^14^ W/cm^2^, i.e. larger red-shaded area in Fig. 1(b3) compared to Fig. 1(a3). These differences are consistent with electron 2 ionizing around the second extremum of the laser field in slingshot-NSDI at 5 × 10^14^ W/cm^2^ and around mostly the first extremum in RESI at 7 × 10^14^ W/cm^2^.

As we increase the laser intensity, it is more probable that the force exerted on electron 2 from the laser field is larger than the attractive Coulomb force exerted from the nucleus. As a result, in most delayed double ionization events, electron 2 does not return at time ${{\rm{t}}}_{2}^{{\rm{ret}}}$ close to the nucleus and therefore does not undergo Coulomb slingshot. That is, following the transition of electron 2 to an excited state, the effect of the nucleus on the motion of this electron decreases with increasing intensity. This results in RESI becoming more important than slingshot-NSDI with increasing intensity.

### Two-electron momentum probability distributions

**Figure 2:**
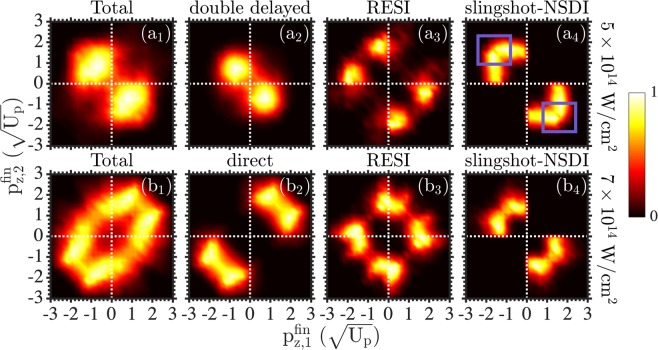
Correlated electron momenta for NSDI pathways. We plot the correlated electron momenta at intensities 5 × 10^14^ W/cm^2^ (top row) and 7 × 10^14^ W/cm^2^ (bottom row) for all double ionization events (**a1**) and (**b1**). At 5 × 10^14^ W/cm^2^, we plot the correlated electron momenta for the double delayed (**a2**) and the delayed pathway, which includes RESI (**a3**) and slingshot-NSDI (**a4**). At 7 × 10^14^ W/cm^2^, we plot the correlated electron momenta for the direct (**b2**) and the delayed pathway, which includes RESI (**b3**) and slingshot-NSDI (**b4**).

In Fig. 2, we plot the correlated electron momenta. That is, we plot the double ionization probability as a function of each electron momentum along the direction of the laser field at intensities 5 × 10^14^ W/cm^2^ (top row) and 7 × 10^14^ W/cm^2^ (bottom row). As the intensity decreases, the two-electron escape changes from correlated, with both electrons escaping in the same direction along the laser field (Fig. 2(b1)), to anti-correlated, see Fig. 2(a1). Two are the main reasons for the transition from anti-correlated two-electron escape at 5 × 10^14^ W/cm^2^ to correlated one at 7 × 10^14^ W/cm^2^. At both intensities we find that two pathways of NSDI prevail, with the delayed pathway being one of the two most important pathways in both cases. However, the other prevailing pathway is the direct one at the higher intensity while it is the double delayed one at the lower intensity. In the direct pathway, both electrons ionize soon after recollision around a zero of the laser field. As a result, the two electrons escape with large final electron momenta, roughly given by −${\mathscr A}({{\rm{t}}}_{{\rm{rec}}})$, in the same direction along the laser field, see Fig. 2(b2). In the double delayed pathway, the two electrons ionize with a delay after recollision resulting in smaller momenta along the laser field compared to direct events. In the double delayed events, the electrons ionize mostly in opposite directions, see Fig. 2(a2).

In addition, as the intensity decreases from 7 × 10^14^ W/cm^2^ to 5 × 10^14^ W/cm^2^, the mechanism underlying the delayed pathway changes from RESI to slingshot-NSDI. At 7 × 10^14^ W/cm^2^, once electron 2 transitions to an excited state of the remaining ion, the electron subsequently ionizes mostly due to the laser field around extrema of the field. As a result electron 2 escapes with a small final momentum. The electron that ionizes first escapes with large momentum, roughly equal to −${\mathscr A}({{\rm{t}}}_{{\rm{rec}}})$. Indeed, this is the pattern exhibited by the correlated electron momenta for RESI both at 5 × 10^14^ W/cm^2^ and at 7 × 10^14^ W/cm^2^, see Fig. 2(a3,b3). At 5 × 10^14^ W/cm^2^, through Coulomb slingshot, the nucleus plays a significant role in ionizing electron 2 after it transitions to an excited state. This effect results in both electrons escaping with large momenta in opposite directions along the laser field, see the two-electron probability distribution enclosed by the squares in Fig. 2(a4). The role of the nucleus diminishes with increasing intensity even for slingshot-NSDI events. Indeed, at the higher intensity, for slingshot-NSDI events, electron 2 does not have quite as large momentum as the first to ionize electron, see Fig. 2(b4) and compare with Fig. 2(a4).

**Figure 3:**
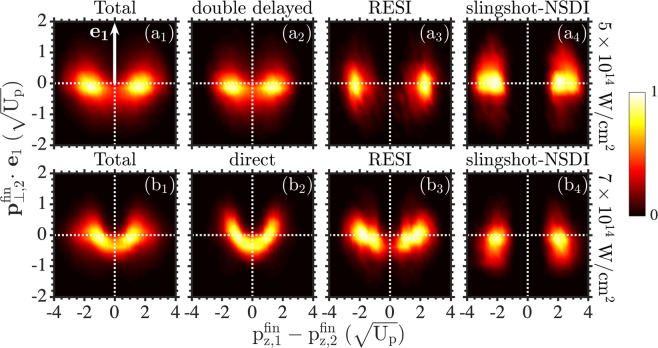
Two-electron momenta distributions. The horizontal axis corresponds to the difference of the electron momenta along the laser field. The vertical axis is along the direction of the perpendicular to the laser field momentum of one of the two electrons, depicted by *e*_1_ in (**a1**). The vertical axis corresponds to the projection of the perpendicular momentum of the other electron on *e*_1_. We plot this two-electron probability distributions for all NSDI events as well as for different NSDI pathways at 5 × 10^14^ W/cm^2^ (top row) and 7 × 10^14^ W/cm^2^ (bottom row), as in Fig. 2.

In Fig. 3, we plot the double ionization probability as a function of the difference of the electron momenta along the laser field and of the projection of the perpendicular momentum of one electron along the direction of the perpendicular momentum of the other electron, denoted by *e*_1_ in Fig. 3(a1). At 7 × 10^14^ W/cm^2^, Fig. 3(b1) shows that for NSDI events with similar electron momenta along the laser field, i.e. ${{\rm{p}}}_{z,1}^{{\rm{fin}}}-{{\rm{p}}}_{z,2}^{{\rm{fin}}}\approx 0$, electron-electron repulsion results in the two electrons escaping with opposite momenta in the direction perpendicular to the laser field. This pattern is due to the direct pathway of NSDI, as Fig. 3(b2) clearly shows. In contrast, electron-electron repulsion does not significantly contribute to RESI and slingshot-NSDI. This is clearly seen in Fig. 3(b3) (RESI) and (b4) (slingshot-NSDI) at 7 × 10^14^ W/cm^2^ and in Fig. 3(a3) (RESI) and (a4) (slingshot-NSDI) at 5 × 10^14^ W/cm^2^. At the lower intensity, a comparison of Fig. 3(a2–a4) shows that electron-electron correlation plays a more important role for the double delayed events rather than for the RESI and the slingshot-NSDI events. This is consistent with both electrons ionizing with a delay in the double delayed events. The authors in ref. ^21^, use a similar two-electron distribution for all double ionization events for He driven by long laser pulses at small intensities at 400 nm. They find that electron-electron repulsion is smaller for anti-correlated two-electron escape rather than for a correlated one. This is consistent with our finding that electron-electron correlation is larger for direct events compared to RESI and slingshot-NSDI events.

### Two-electron angular probability distributions

**Figure 4:**
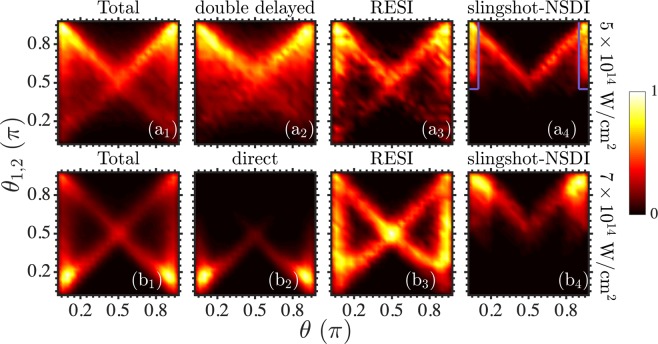
Two-electron angular probability distributions. We plot the double ionization probability as a function of the inter-electronic angle *θ*_1,2_ and as a function of the angle *θ* at intensities 5 × 10 ^14^ W/cm^2^ (top row) and 7 × 10^14^ W/cm^2^ (bottom row) for all double ionization events (**a1**) and (**b1**) and for the dominant NSDI pathways as in Fig. 2.

In Fig. 4, we plot the double ionization probability as a function of the inter-electronic angle between the momenta of the two escaping electrons and the angle formed by the momentum of one of the two electrons with respect to the axis of the laser field. Comparing Fig. 4(a1,b1), we find that for most double ionization events the inter-electronic angle is close to 180° at 5 × 10^14^ W/cm^2^ and close to 0° at 7 × 10^14^ W/cm^2^. This is consistent with anti-correlated two-electron escape prevailing at 5 × 10^14^ W/cm^2^ and correlated prevailing at 7 × 10^14^ W/cm^2^, as we have already seen in Fig. 2(a1,b1), respectively. At 5 × 10^14^ W/cm^2^, Fig. 4(a1) shows that for most double ionization events, in addition to the two electrons escaping opposite to each other, one of the two electrons escapes along the polarization direction, i.e. *θ* is 0° or 180°. This latter pattern of two-electron escape is more pronounced for slingshot-NSDI, see Fig. 4(a4) and compare with Fig. 4(a2,a3). We find that the electron escaping along the polarization axis is the one that ionizes first after recollision, giving rise to the probability distribution enclosed by the parallelograms in Fig. 4(a4). The remaining wedge-like shape in Fig. 4(a4) is accounted for by electron 2 forming an angle *θ* with the polarization axis while, for *θ* ∈[0°, 90°], electron 1 forms an angle 180° giving rise to *θ*_1,2_ = 180° − *θ* and, for *θ* ∈[90°, 180°], electron 1 forms an angle 0° giving rise to *θ*_1,2_ = *θ*. At the higher intensity, the prevailing correlated two-electron escape is mostly due to the direct pathway of double ionization, see Fig. 4(b2) and compare with Fig. 4(b3,b4).

### Two-electron energy probability distributions

**Figure 5:**
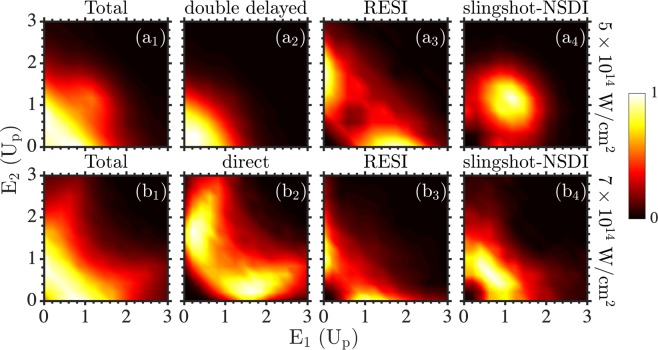
Two-electron energy probability distributions. We plot the double ionization probability as a function of the energies of the two escaping electrons expressed in U_p_ at laser intensities 5 × 10^14^ W/cm^2^ (top row) and 7 × 10^14^ W/cm^2^ (bottom row) for all double ionization events (**a1**) and (**b1**) and for the dominant NSDI pathways as in Fig. 2.

Besides anti-correlated two-electron escape, we find that another hallmark of slingshot-NSDI is both electrons escaping with large energy. This is clearly seen at the smaller intensity of 5 × 10^14^ W/cm^2^ in Fig. 5(a4). In contrast, RESI gives rise to an unequal energy sharing between the two escaping electrons. This can be seen at 5 × 10^14^ W/cm^2^ in Fig. 5(a3) and at 7 × 10^14^ W/cm^2^ in Fig. 5(b3). Both in RESI and slingshot-NSDI electron 1 escapes with large energy. This is mainly due to the large momentum of electron 1 along the direction of the laser field at the recollision time, which is roughly equal with −${\mathscr A}({{\rm{t}}}_{{\rm{rec}}})$. The main difference between RESI and slingshot-NSDI is the influence of the nucleus on electron 2 following its transition to an excited state after recollision. For RESI, the nucleus has a very small effect on electron 2. This electron ionizes with the help of the laser-field around field extrema, resulting in mostly small final energy of electron 2. As a result, unequal energy sharing prevails in RESI, see Fig. 5(a3,b3). In contrast, in slingshot-NSDI the nucleus plays a major role on electron 2. As we have previously discussed, this electron undergoes a Coulomb slingshot motion around the nucleus gaining a large amount of energy. As a result, roughly equal energy sharing prevails in slingshot-NSDI, see Fig. 5(a4). This pattern gives rise to a high concentration of NSDI probability around the diagonal at large energies in the two-electron energy probability distribution for all NSDI events, see Fig. 5(a1).

The effect of the nucleus on electron 2, following its transition to an excited state, decreases with increasing intensity. This is manifested in RESI overtaking slingshot-NSDI as the prevailing mechanism of the delayed pathway. This diminishing effect of the nucleus on electron 2 in the delayed pathway is also evident in slingshot-NSDI events. Indeed, the two electrons share the energy more unequally at 7 × 10^14^ W/cm^2^ in Fig. 5(b4) compared to 5 × 10^14^ W/cm^2^ in Fig. 5(a4). The reduced contribution of slingshot-NSDI to the delayed pathway as well as the reduced effect of the nucleus on electron 2 in slingshot-NSDI at 7 × 10^14^ W/cm^2^, accounts for the more unequal energy sharing of the two electrons for all NSDI events in Fig. 5(b1) versus more equal energy sharing at 5 × 10^14^ W/cm^2^ in Fig. 5(a1).

## Conclusions

Using a 3D semiclassical model we investigate how two-electron probability distributions change for different NSDI pathways as well as for different intensities below-the-recollision-threshold. We do so for He driven by near-single-cycle laser pulses at 400 nm. This study allows us to gain significant insight into slingshot-NSDI, which is a new mechanism that prevails the delayed pathway of NSDI at low intensities, as we have recently shown^29^. We find that in slingshot-NSDI the electron that ionizes first does so along the polarization direction of the laser field. The electron that ionizes last escapes opposite to the other electron. This is due to electron 2 undergoing a Coulomb slingshot motion around the nucleus. Indeed, we have shown that, following recollision, unlike direct events of NSDI where electron-electron correlation has a significant effect, electron repulsion has a very small effect on both RESI and slingshot-NSDI. Moreover, we find that in slingshot-NSDI the two electrons escape both with large energy giving rise to a distinct equal energy pattern in the two-electron energy probability distribution for all double ionization events. Thus, in addition to the anti-correlated two-electron escape, which we have previously identified as a hallmark of slingshot-NSDI^29^, two-electron escape with roughly equal energy sharing and large energies is yet another trademark of slingshot-NSDI which can be measured by future experiments.
